# Time to Learn: The Role of the Clock Gene Per1 in Regulating Memory Across the Lifespan

**DOI:** 10.1093/geroni/igaf122.680

**Published:** 2025-12-31

**Authors:** Janine Kwapis

**Affiliations:** Pennsylvania State University, University Park, Pennsylvania, United States

## Abstract

Many biological processes, including memory formation, are strongly influenced by the circadian system, which synchronizes animals’ internal states with the external time of day. Long-term memory performance changes across the day/night cycle in both humans and rodents, yet the mechanisms that support this process are largely unknown. In this talk, I will present my lab’s research suggesting that a circadian clock gene, Period1 (Per1) may serve as a molecular interface between the circadian clock and memory formation. Per1 oscillates in tandem with memory in memory-relevant brain regions, like the hippocampus, with both memory and Per1 levels peaking during the day and showing a trough at night. Interestingly, old animals have repressed hippocampal Per1 levels even during the day, suggesting that they may show impairments in memory due to this persistent “nighttime state” that limits memory across the diurnal cycle. Finally, we find that young and old female mice show unique memory oscillation patterns that depend on sex hormone expression. Together, our data suggest that Per1 may function locally in memory-relevant brain regions to exert diurnal control over memory and age-related disruption of Per1 may contribute to age-related cognitive decline.

